# Structural mechanism of allosteric activation of TRPML1 by PI(3,5)P_2_ and rapamycin

**DOI:** 10.1073/pnas.2120404119

**Published:** 2022-02-07

**Authors:** Ninghai Gan, Yan Han, Weizhong Zeng, Yan Wang, Jing Xue, Youxing Jiang

**Affiliations:** ^a^HHMI, University of Texas Southwestern Medical Center, Dallas, TX 75390-9040;; ^b^Department of Physiology, University of Texas Southwestern Medical Center, Dallas, TX 75390-9040;; ^c^Department of Biophysics, University of Texas Southwestern Medical Center, Dallas, TX 75390-9040

**Keywords:** TRPML1, rapamycin, PI(3,5)P_2_, lysosomal channel

## Abstract

Transient receptor potential mucolipin 1 (TRPML1) is a Ca^2+^-permeable, nonselective cation channel ubiquitously expressed in the endolysosomes of mammalian cells and its loss-of-function mutations are the direct cause of type IV mucolipidosis (MLIV), an autosomal recessive lysosomal storage disease. TRPML1 is a ligand-gated channel that can be activated by phosphatidylinositol 3,5-bisphosphate [PI(3,5)P_2_] as well as some synthetic small-molecule agonists. Recently, rapamycin has also been shown to directly bind and activate TRPML1. Interestingly, both PI(3,5)P_2_ and rapamycin have low efficacy in channel activation individually but together they work cooperatively and activate the channel with high potency. To reveal the structural basis underlying the synergistic activation of TRPML1 by PI(3,5)P_2_ and rapamycin, we determined the high-resolution cryoelectron microscopy (cryo-EM) structures of the mouse TRPML1 channel in various states, including apo closed, PI(3,5)P_2_-bound closed, and PI(3,5)P_2_/temsirolimus (a rapamycin analog)-bound open states. These structures, combined with electrophysiology, elucidate the molecular details of ligand binding and provide structural insight into how the TRPML1 channel integrates two distantly bound ligand stimuli and facilitates channel opening.

## Significance

Rapamycin is a specific inhibitor of mammalian target of rapamycin (mTOR). Rapamycin can also activate transient receptor potential mucolipin 1 (TRPML1), a phosphatidylinositol 3,5-bisphosphate [PI(3,5)P_2_]–gated lysosomal cation channel whose loss-of-function mutations directly cause mucolipidosis type IV disease. We determined the high-resolution cryoelectron microscopy structures of TRPML1 in various ligand-bound states, including the open TRPML1 in complex with PI(3,5)P_2_ and a rapamycin analog at 2.1 Å. These structures reveal how rapamycin and PI(3,5)P_2_ bind at two distinct sites and allosterically activate the channel. Considering the high potency of TRPML1 activation by rapamycin and PI(3,5)P_2_, it is conceivable that some pharmacological effects from the therapeutic use of rapamycin may come from the TRPML1-dependent mechanism rather than mTOR inhibition.

Rapamycin (also known as sirolimus) is a natural macrocyclic lactone initially isolated from *Streptomyces hygroscopicus* (1). Mammalian target of rapamycin (mTOR), a serine/threonine kinase highly conserved in eukaryotes and predominantly localized to lysosomal membranes under nutrient-rich conditions, is the first identified physical target of rapamycin (2–4). mTOR can interact with multiple proteins to form mTOR complex 1 (mTORC1) or complex 2 (mTORC2). mTORC1 is a critically important signal integrator that regulates multiple cellular events including synthesis of lipids, proteins, and nucleotides, energy metabolism, nutrient sensing, and autophagy (5). Rapamycin functions as an allosteric inhibitor of mTORC1. It forms a ternary FKBP12–rapamycin–mTORC1 complex by binding to the immunophilin FK506-binding protein 12 (FKBP12) and the rapamycin-binding domain of mTOR, thereby preventing the recruitment of mTOR substrates and inhibiting mTOR signaling (6, 7). Given the vital role of mTOR in cell proliferation and growth, its inhibition by rapamycin exhibits great anticancer and immunosuppressive effects. Indeed, rapamycin and its derivatives have been developed for the prevention of transplant rejection and the treatment of cancer and multiple metabolic and neurodegenerative diseases (8, 9). Because of the therapeutic importance of rapamycin and its analogs, most rapamycin-related studies have been focused on its inhibition of mTOR-dependent signaling. Whether rapamycin could also have an mTOR-independent pharmacological effect by targeting proteins other than mTOR has not been well-studied. Recently, it was reported that rapamycin and its derivatives can directly bind and activate transient receptor potential mucolipin 1 (TRPML1) in an mTOR-independent manner (10).

TRPML1 is a Ca^2+^-permeable, nonselective, six-transmembrane tetrameric cation channel ubiquitously expressed in mammalian cells and is primarily localized to the endolysosomal membrane. The lysosome is the center for recycling and degradation of biological materials and also serves as one of the major intracellular Ca^2+^ stores (11, 12). TRPML1 has been suggested to be the major calcium-releasing channel in lysosomes and is involved in multiple important cellular activities including lysosome trafficking, lipid accumulation, signaling transduction, and autophagy (13–16). Loss-of-function mutations in human TRPML1 are the direct cause of the lysosomal storage disorder mucolipidosis type IV, a neurodegenerative disease characterized by abnormal neurodevelopment, retinal degeneration, and iron-deficiency anemia (17–20). Furthermore, TRPML1 function is also compromised in the cells of Niemann–Pick disease type C (14). TRPML1 can be activated and modulated by both natural and synthetic ligand molecules (14, 21–24). Endogenously, TRPML1 can be activated by the lysosome-specific phosphatidylinositol 3,5-bisphosphate [PI(3,5)P_2_] (25) but inhibited by the plasma membrane–enriched PI(4,5)P_2_ (26). However, the PI(3,5)P_2_-activated TRPML1 channel has low single-channel activity (26, 27) and, consistent with this functional observation, the structure of PI(3,5)P_2_-bound TRPML1 remains in the closed conformation (28). It is possible that some other cellular factors also participate in the activation or modulation of TRPML1. Similar to PI(3,5)P_2_, rapamycin by itself also has very low potency of TRPML1 activation. Interestingly, rapamycin and PI(3,5)P_2_ together can work cooperatively and activate the channel with high potency (10). In this study, we used temsirolimus (Tem; a rapamycin analog) in place of rapamycin and functionally confirmed the synergistic activation of TRPML1 by PI(3,5)P_2_ and Tem. We also determined the structures of the mouse TRPML1 channel in the apo closed, PI(3,5)P_2_-bound closed, PI(3,5)P_2_/Tem-bound open, and mucolipin synthetic agonist 1 (ML-SA1)–bound open states. All determined at high resolution, these structures provide clear visualization of the molecular details of protein–ligand interactions in TRPML1 and reveal the structural basis underlying the synergistic gating of TRPML1 by rapamycin and PI(3,5)P_2_.

## Results

### Electrophysiology of TRPML1 Activation.

For electrophysiological analysis, the two dileucine motifs (_15_LL and _577_LL) of mouse TRPML1 responsible for lysosomal targeting were replaced with alanines to facilitate the trafficking of the channel to the plasma membrane (22, 29). The mutant channel, TRPML1-4A, was overexpressed in HEK293 cells and its activity was measured by patching the plasma membrane. In this setting, the extracellular side is equivalent to the luminal side of TRPML1 in endo/lysosomes. Tem, a rapamycin analog sharing a very similar chemical structure and channel activation property to rapamycin (10) (*SI Appendix*, Fig. S1), was used in our functional as well as structural analysis. The Tem activation of the channel was compared with that of other commonly used TRPML1 agonists, including PI(3,5)P_2_ and ML-SA1.

**Fig. 1.**
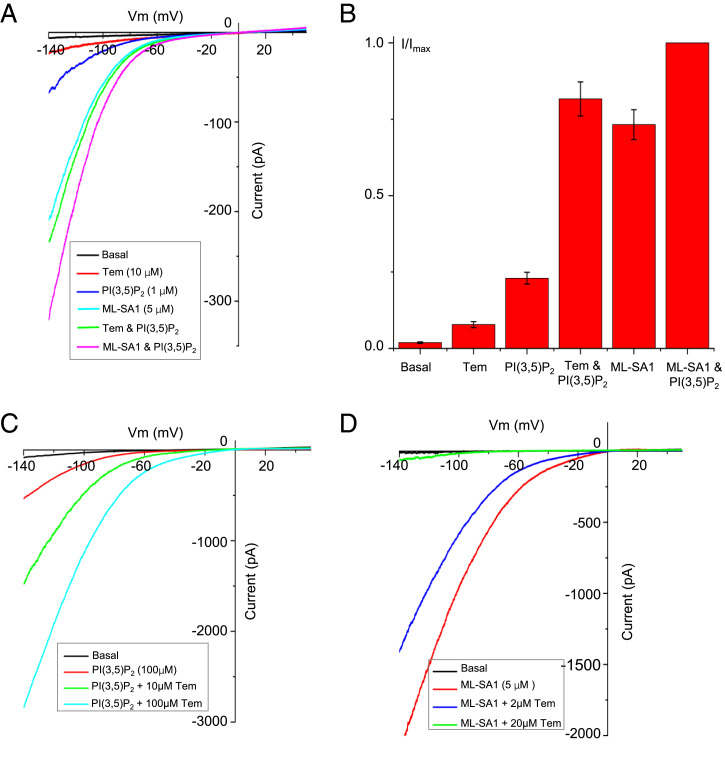
Electrophysiology of TRPML1 activation. (*A*) Sample traces of TRPML1 activation recorded using a patch clamp in inside-out configuration with various ligands introduced into the bath solution (cytosolic). All traces were obtained from the same patch. The same ligand concentration was used when applied individually or as a mixture. (*B*) Comparison of TRPML1 currents at −140 mV elicited by the individual or mixed ligands shown in *A*. Currents are normalized against the current elicited by the mixed ligands of ML-SA1 (5 µM) and PI(3,5)P_2_ (1 μM). Data points are mean ± SEM (*n* = 5 independent experiments). (*C*) Sample traces of TRPML1 activation recorded using a patch clamp in whole-cell configuration with 100 µM PI(3,5)P_2_ in the pipette (cytosolic). Tem was introduced into the bath solution (extracellular/luminal). The basal current was recorded right after membrane breakthrough. The PI(3,5)P_2_-activated current was recorded about 3 min after membrane breakthrough, allowing the lipid ligand to diffuse into the cell and yield a stable current. (*D*) Inhibition of ML-SA1 activation by Tem recorded using a patch clamp in whole-cell configuration in the absence of PI(3,5)P_2_. ML-SA1 and Tem were both introduced into the bath solution (extracellular/luminal).

As shown in the recordings of TRPML1 using inside-out patches with various agonists added to the bath solution (cytosolic side) (Fig. 1*A*), both PI(3,5)P_2_ and ML-SA1 can activate the TRPML1 channel individually and also synergistically when present together. PI(3,5)P_2_ alone has a much lower potency of TRPML1 activation than ML-SA1. This is consistent with previous studies demonstrating that PI(3,5)P_2_-activated TRPML1 has a low single-channel open probability (26, 27). Interestingly, Tem only weakly activates the channel by itself but can elicit much larger inwardly rectifying currents when present together with PI(3,5)P_2_. This strong synergistic activation of TRPML1 by PI(3,5)P_2_ and Tem is comparable to that by PI(3,5)P_2_ and ML-SA1 (Fig. 1 *A* and *B*).

In the presence of cytosolic PI(3,5)P_2_, Tem can also potently activate the channel from the extracellular side of the membrane (equivalent to the luminal side of the lysosome) as illustrated in the recordings using whole-cell patches (Fig. 1*C*). The ability of Tem to activate TRPML1 from both sides suggests that this lipophilic agonist likely targets the channel from within the membrane. Indeed, as shown later in the PI(3,5)P_2_/Tem-bound TRPML1 structure, the Tem-binding site is located in the middle of the membrane and overlaps with that of the ML-SA1 agonist. Because Tem is a much weaker channel activator by itself, this overlapped binding enables Tem to competitively inhibit ML-SA1 activation in the absence of PI(3,5)P_2_ (Fig. 1*D*).

### Structural Determination of TRPML1 with Various Ligands.

The cryoelectron microscopy (cryo-EM) structures of mouse TRPML1 were determined by using protein samples prepared in the presence of PI(3,5)P_2_ and/or Tem (*SI Appendix*, Figs. S2–S6 and Table S1). Our initial goal was to obtain the structures of TRPML1 in complex with individual and both ligands. However, particles from the protein sample prepared in the presence of Tem alone were mostly unligated, likely because of low-affinity Tem binding, yielding an apo closed TRPML1 structure at 2.6-Å resolution (*SI Appendix*, Fig. S2). This is consistent with the functional observation of weak TRPML1 activation by Tem alone. Nevertheless, this apo structure has a much higher resolution than previously reported ones and therefore provides a better model for structural comparison in this study.

The particles from the protein sample prepared in the presence of PI(3,5)P_2_, on the other hand, are mostly ligated, yielding a PI(3,5)P_2_-bound TRPML1 structure at 2.6-Å resolution (*SI Appendix*, Fig. S3). The density for the inositol 1,3,5-trisphosphate (IP3) head group of the lipid is well-defined in the EM map whereas the density for the flexible fatty acid chain is poorly resolved. Despite the presence of ligand, the PI(3,5)P_2_-bound TRPML1 structure remains in the closed conformation, as was observed previously (28).

There are two major classes of particles in the protein sample prepared in the presence of both PI(3,5)P_2_ and Tem. In one class, the channel adopts an open conformation with both ligands bound, and the structure was refined to 2.1 Å; in the other class, the channel remains in the closed conformation with only PI(3,5)P_2_ bound, and the structure was refined to 2.4 Å (*SI Appendix*, Fig. S4). The latter structure is virtually identical to that obtained from the sample prepared in the presence of PI(3,5)P_2_ alone but has a higher resolution, and therefore is used as the model for the PI(3,5)P_2_-bound closed TRPML1 in this study. The synthetic agonist ML-SA1 can by itself stabilize the TRPML1 channel in an open conformation and the structure of the channel in complex with ML-SA1 was determined at low resolution (∼3.5 Å) in a previous study (30). For a better comparison with the PI(3,5)P_2_/Tem-bound structure, we also determined the ML-SA1–bound open TRPML1 structure at 2.3-Å resolution using our protein sample prepared in the presence of this synthetic agonist (*SI Appendix*, Fig. S5).

### Structures of Closed TRPML1 in Apo and PI(3,5)P_2_-Bound States.

**Fig. 2.**
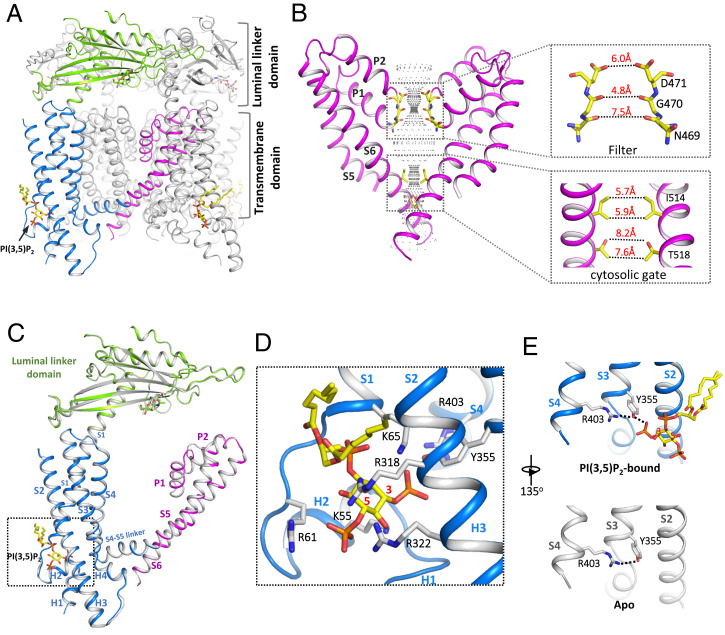
Structure of closed TRPML1 in apo and PI(3,5)P_2_-bound states. (*A*) Side view of a cartoon representation of the PI(3,5)P_2_-bound closed TRPML1 structure with the three major domains from the front subunit colored (blue for S1 to S4, green for the luminal linker domain, and magenta for the pore domain). (*B*) The closed ion-conduction pore of TRPML1 with only two diagonal subunits shown for clarity. The central ion pathway is marked with dotted mesh. Key gating and filter residues are shown as sticks. (*Insets*) Zoomed-in views of the filter and the cytosolic gate. (*C*) Structural comparison of a single subunit between apo (gray) and PI(3,5)P_2_-bound (colored) TRPML1. (*D*) Zoomed-in view of the PI(3,5)P_2_-binding site with key ligand-interacting residues shown. The red numbers mark the C3 and C5 positions of inositol. (*E*) H-bonding interaction between R403 and Y355 in the presence and absence of PI(3,5)P_2_.

The binding of PI(3,5)P_2_ alone is insufficient to stabilize the channel in the open conformation and the PI(3,5)P_2_-bound TRPML1 structures adopt the same closed conformation as the apo structure (Fig. 2). This is consistent with the functional analysis of TRPML1 showing low channel open probability when activated by PI(3,5)P_2_. The closed ion-conduction pore of TRPML1 contains two constrictions along the ion pathway: One is formed by the carbonyl oxygen atoms of Gly470s at the filter region with a diagonal atom-to-atom distance of about 4.8 Å; the other is formed by the hydrophobic side chains of Ile514s with a diagonal atom-to-atom distance of about 5.7 Å and serves as the cytosolic gate of the channel (Fig. 2*B*).

Determined at a much higher resolution than the previously reported one (28), the current PI(3,5)P_2_-bound TRPML1 structure provides a more accurate view of the lipid position and its interaction with the channel. PI(3,5)P_2_ binds in a pocket enclosed by two short clamp-shaped helices of H1 and H2 right before the transmembrane S1 helix and the cytosolic ends of the S1 and S2 helices. Multiple positively charged amino acids participate in the interactions with the IP3 head group (Fig. 2 *C* and *D*). Other than small local structural changes to accommodate the lipid at the ligand-binding site, the TRPML1 structures with or without PI(3,5)P_2_ are almost identical (Fig. 2*C*). It is worth noting that an H-bonding triad formed by R403, Y355, and the C3 phosphate group has been suggested to be important for PI(3,5)P_2_ activation (Fig. 2*E*) (28). It was proposed that PI(3,5)P_2_ may activate the channel by promoting a π-cation interaction between R403 and Y355. However, no π-cation interaction is observed in our apo or PI(3,5)P_2_-bound closed structure, nor in the PI(3,5)P_2_/Tem-bound open structure discussed below. R403 and Y355 maintain a similar H-bonding interaction in all these structures regardless of ligation state (Fig. 2*E*).

### Structure of Open TRPML1 in Complex with PI(3,5)P_2_ and Tem.

**Fig. 3.**
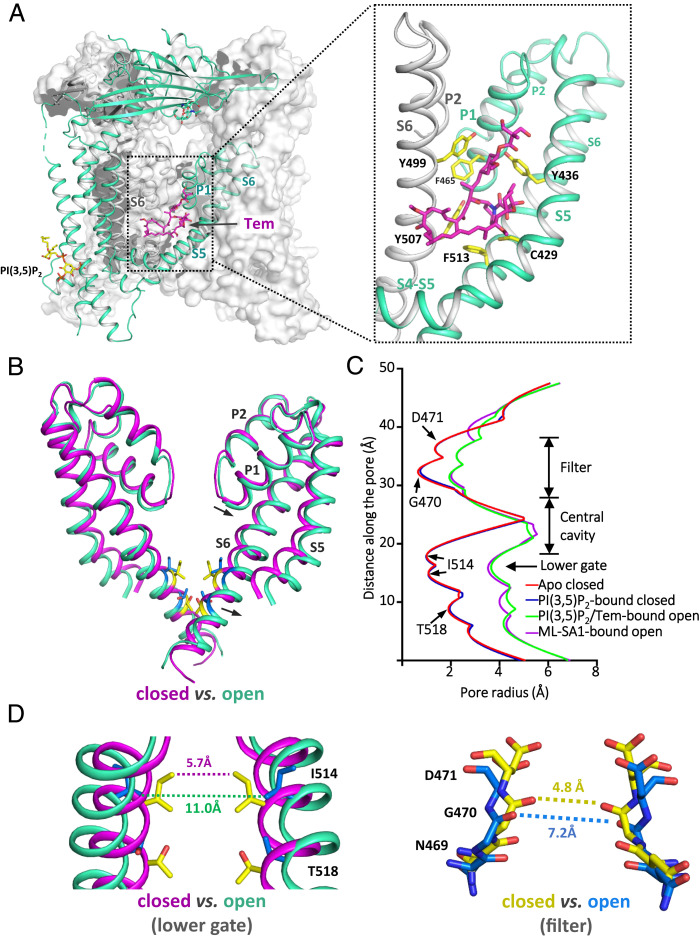
Structure of TRPML1 in the PI(3,5)P_2_/Tem-bound open state. (*A*) Structure of PI(3,5)P_2_/Tem-bound TRPML1 with the front subunit shown as a green cartoon and the rest shown in gray surface representation. (*Inset*) Zoomed-in view of the Tem-binding site between S5 (green) and S6 (gray) of the neighboring subunit. Side chains of ligand-interacting residues are shown as yellow sticks. (*B*) Structural comparison of the ion-conduction pore between the closed (magenta) and open (green) states. Arrows indicate the movements from the closed to open state. (*C*) Pore radius along the central axis in the open and closed states. (*D*) Structural comparison of the lower gate and the selectivity filter between the open and closed states.

The structure of TRPML1 in complex with both PI(3,5)P_2_ and Tem adopts an open conformation with well-defined density for the bound PI(3,5)P_2_ and Tem (Fig. 3 and *SI Appendix*, Fig. S6). PI(3,5)P_2_ in this open structure has the same configuration and engages in similar interactions with the channel as that in the PI(3,5)P_2_-bound closed channel. One key difference between them is the formation of an extra salt bridge between PI(3,5)P_2_ and Arg403 in the open structure, as will be discussed further below. Embedded almost in the middle of the membrane, Tem binds at an intersubunit interface between S5 and S6 from two neighboring subunits, respectively (Fig. 3*A*). This binding site appears to be a hotspot for both channel agonist and antagonist, as it overlaps with the sites for ML-SA1 and synthetic antagonist ML-SI3 (*SI Appendix*, Fig. S7*A*).

In the open TRPML1, both constrictions observed in the closed pore become widened (Fig. 3 *B*–*D*). The opening of the lower gate is achieved by a quite subtle outward tilt of S6 helices hinged at its N terminus, allowing the constriction-forming Ile514 to dilate away from the central axis (Fig. 3*D* and Movie S1). Consequently, the minimum distance at the cytosolic gate expands from 5.7 to 11.0 Å (atom-to-atom distance). The filter conformational change is coupled to the S6 movement owing to the tight packing between the two pore helices (P1 and P2) and S6 at the periphery of the filter. As a result, the tilt movement of S6 upon channel activation also drags the pore helices and the filter along with it, causing about 2.4-Å expansion in filter diameter at the constriction-forming Gly470 (Fig. 3*D* and Movie S2). Similar pore-opening mechanics was also observed in ML-SA1–activated TRPML1 and TRPML3 structures (30, 31).

### Allosteric Activation of TRPML1 by PI(3,5)P_2_ and Tem.

**Fig. 4.**
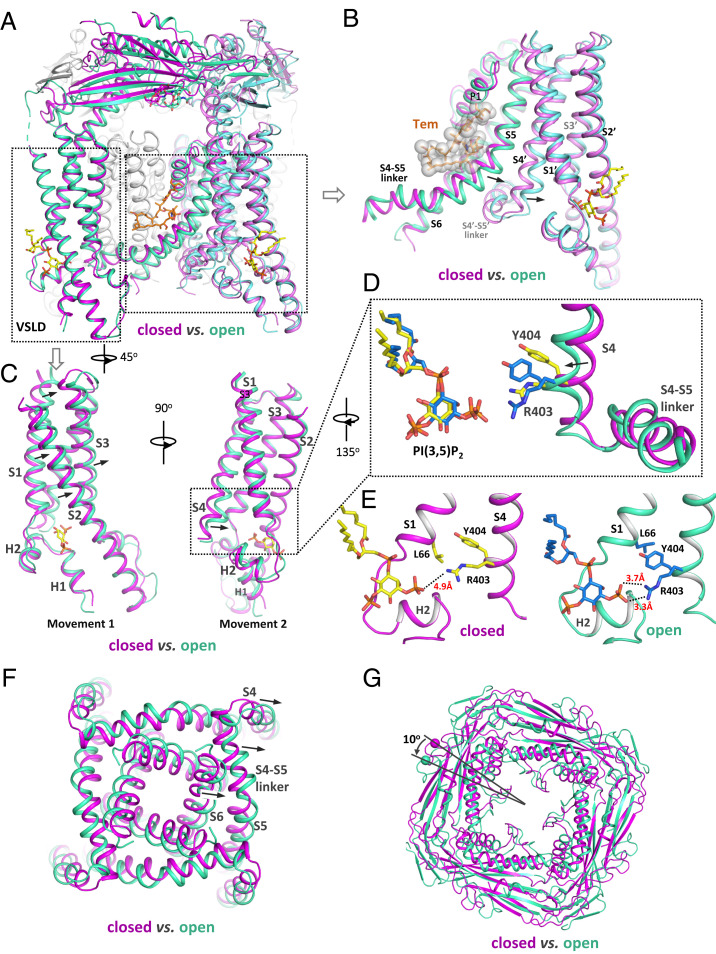
Conformational changes of TRPML1 between the PI(3,5)P_2_-bound closed and PI(3,5)P_2_/Tem-bound open states. (*A*) Structural comparison of TRPML1 between the PI(3,5)P_2_-bound closed (magenta) and PI(3,5)P_2_/Tem-bound open (green) states. (*B*) Conformational changes at the Tem-binding region. Arrows indicate the Tem-induced movements at S5 and S4 from the neighboring subunit (labeled as S4′). (*C*) Two subtle movements at the PI(3,5)P_2_-binding region. Arrows indicate the movements from the closed to open state. (*D*) Zoomed-in view of the R403/Y404-mediated bending movement of S4. (*E*) Zoomed-in view of R403/Y404-mediated interactions that are observed in the open state (*Right*) but absent in the closed state (*Left*). (*F*) Top view of the pore-opening movement induced by the outward movement of the S4 C terminus. (*G*) Top view of the counterclockwise rotation of the luminal linker domain from the closed to open state.

The structural comparison between the open [in complex with PI(3,5)P_2_ and Tem] and closed [in complex with PI(3,5)P_2_ alone] states reveals how TRPML1 integrates the two ligand stimuli and facilitates the channel opening (Fig. 4*A*). At the Tem site, the N-terminal part of the S5 helix bulges outwardly to expand the groove space between S5 and S6 to accommodate the binding of the bulky ring-structured agonist. With tight intersubunit interactions between S5 and S4 from the neighboring subunit (labeled as S4′ in Fig. 4*B*), this S5 movement would directly push the C-terminal part of S4, forcing it to also move outwardly and toward the PI(3,5)P_2_ site (Fig. 4*B* and Movie S3).

The local conformational changes from closed to open state at the PI(3,5)P_2_-binding site are subtle but more complex and likely consist of two sequential movements (Fig. 4*C*). First, the S1–S4 voltage sensor–like domain (VSLD) along with the bound PI(3,5)P_2_ undergoes a slightly up- and rightward movement tangential to the pore (Fig. 4*C* and Movie S4). The second movement involves a slight bend of the C-terminal half of the S4 driven by the thrust of the Tyr404 side chain into a pocket located between the S1 and S3 helices and above the bound PI(3,5)P_2_, resulting in the tilt of the S4 C terminus away from the central pore (Fig. 4 *C* and *D* and Movie S5). Two interactions stabilize the bent S4 in the open conformation: The extended side chain of Arg403 moves closer to the head group of the bound PI(3,5)P_2_ and forms a salt bridge with its C3 phosphate; the aromatic ring of Tyr404 in the pocket is sandwiched between the side chains of Leu66 and Arg403 (Cβ-to-Cδ portion) and stabilized by hydrophobic packing (Fig. 4*E*). As expected, both Arg403 and Tyr404 are highly conserved in the TRPML channel family. The first movement appears to be a prerequisite for the second one, as it releases the steric hindrance that prevents the insertion of Tyr404 into the pocket. In essence, the outcome of the local conformational change driven by PI(3,5)P_2_ binding is analogous to the pulling of the C-terminal part of S4 toward PI(3,5)P_2_.

The local conformational changes induced by the two distantly bound ligands both converge to the same driving force on the S4 helix, causing its C terminus to depart away from the pore. Through direct linkage, the S4–S5 linker undergoes the same outward movement along with S4. As the four S4–S5 linker helices in a channel tetramer cuff around the pore-lining S6 helices with extensive hydrophobic interactions, their outward movements are directly coupled to the bending of the S6 helices, resulting in the opening of the pore (Fig. 4*F* and Movie S1). Thus, S4 is the central hub in TRPML1 that integrates the driving forces from two distantly bound ligand stimuli and mediates their allosteric activation of the channel: Upon Tem binding, the expansion of S5 would impose a pushing force onto S4, allowing it to move toward the PI(3,5)P_2_ site and facilitate the PI(3,5)P_2_-driven conformational changes; likewise, the pulling of S4 upon PI(3,5)P_2_ binding is also coupled to S5, whose expansion movement would facilitate Tem binding.

It is interesting to note that the small local conformational changes at both ligand sites propagate to multiple parts of the channel through tight inter- and intrasubunit packing within the channel tetramer, resulting in a global movement of almost the entire channel beyond the pore opening at the S6 helices (Movie S6). For instance, because the large luminal linker domain atop the channel is pillared by the four S1 helices, the rightward shift of S1 upon channel opening also leads to a 10° counterclockwise rotation of the linker domain when viewing from the luminal side (Fig. 4*G* and Movie S7).

## Discussion

Here we present the structural and functional analysis of the allosteric activation of TRPML1 by PI(3,5)P_2_ and the rapamycin analog Tem. With a similar chemical structure and equivalent functional effect on TRPML1, we expect that rapamycin and Tem share the same binding site and activation mechanism. Given the high potency of synergistic activation of TRPML1 by Tem (or rapamycin) and the endogenous PI(3,5)P_2_, it is conceivable that some pharmacological effects from the therapeutic use of rapamycin or its analogs could be partly contributed by TRPML1-dependent mechanisms, such as the promotion of autophagy (10).

Although PI(3,5)P_2_ can readily bind TRPML1, the PI(3,5)P_2_-bound channel has low open probability and remains mostly in the closed conformation, suggesting that PI(3,5)P_2_ binding alone can reduce but not overcome the energy barrier between the closed and open states. Tem also has low efficacy in TRPML1 activation. However, different from PI(3,5)P_2_, Tem does not form a stable complex with TRPML1 by itself, indicating its state-dependent binding with low affinity to the closed channel. When present together, PI(3,5)P_2_ and Tem can synergistically activate TRPML1 by binding at two distant locations and stabilizing the channel in an open conformation. The binding of Tem is only observed in the open channel in the presence of PI(3,5)P_2_, suggesting that PI(3,5)P_2_ binding is a prerequisite for Tem binding and the allosteric channel activation. It is likely that PI(3,5)P_2_ binding increases the chance of channel transition to the open conformation that would allow for Tem binding, which in turn provides further stabilization to the open channel and shifts the equilibrium toward the open state.

The Tem-binding site overlaps with that of the small-molecule agonist ML-SA1, and they appear to exert the same driving force on S5 and likely yield similar local conformational changes. ML-SA1, however, is a highly potent agonist and is sufficient to bind and stabilize the channel in the open conformation by itself (30, 31). In the ML-SA1–bound TRPML1, because of the tight packing and coupling among various parts of the channel, the local conformational change at the ML-SA1 site also propagates to other parts, including the PI(3,5)P_2_ site and the luminal linker domain despite the absence of PI(3,5)P_2_. As a result, the ML-SA1–bound open TRPML1 structure is almost identical to that of the Tem/PI(3,5)P_2_-bound open channel (*SI Appendix*, Fig. S7*B*). This coupled movement is the underlying structural basis for the allosteric regulation of TRPML1 between two distantly bound ligands. That is, the local conformational change driven by one bound ligand can propagate to the other allosteric site and thereby affect the binding of the other ligand. While we see positive cooperativity between PI(3,5)P_2_ and Tem in channel activation, some TRPML1 antagonists can also compete for the same binding sites and exert negative allosteric regulation. For example, the small-molecule antagonist ML-SI3 can compete for the ML-SA1 site (32) and negatively regulate the PI(3,5)P_2_ activation of TRPML1; likewise, PI(4,5)P_2_ can compete for the PI(3,5)P_2_ site and negatively regulate ML-SA1 activation (26). In the structures of the open channel activated by PI(3,5)P_2_ and Tem or by ML-SA1 alone, the luminal linker domain undergoes a similar rotation movement coupled to the shift of the S1 helix. We suspect that any luminal stimulus that inhibits or promotes this rotation movement can also allosterically modulate the channel gating.

## Materials and Methods

### Protein Expression and Structural Determination.

The *Mus musculus* TRPML1 gene with a C-terminal thrombin cleavage site and a 10× His tag was expressed in HEK293F cells at 37 °C for 48 h using the BacMam system. Protein was extracted with 1% (weight/volume [wt/vol]) *n*-dodecyl-β-d-maltopyranoside (Anatrace) supplemented with 0.2% (wt/vol) cholesteryl hemisuccinate (Sigma-Aldrich) or 1% (wt/vol) lauryl maltose neopentyl glycol (Anatrace) by gentle agitation for 2 h. After purification with Ni-NTA resin and size-exclusion chromatography on a Superose 6 10/300 GL column (GE Healthcare), the final sample was concentrated to 3.5 mg/mL for single-particle analysis. The cryo-EM grids were prepared using a Mark IV Vitrobot (FEI). Micrographs were acquired on a Titan Krios microscope (FEI) operated at 300 kV with a K3 Summit direct electron detector (Gatan), using a slit width of 20 eV on a GIF-Quantum energy filter. Data were collected using the correlated double-sampling mode of the K3 camera with a superresolution pixel size of 0.415 Å. The defocus range was set from −0.9 to −2.2 μm. Each movie was dose-fractionated to 60 frames with a dose rate of 1e^−^/Å^2^ per frame for a total dose of 60e^−^/Å^2^. The total exposure time was between 5 and 6 s. Image processing and three-dimensional reconstruction were performed following standard procedures, and the final resolution was estimated by the gold-standard Fourier shell correlation = 0.143 criterion (*SI Appendix*, Figs. S2–S5 and Table S1). The structure of mouse TRPML1 (Protein Data Bank [PDB] ID code 5WPV) was used as the initial model for model building.

### Electrophysiology.

The N-terminal green fluorescent protein–tagged, plasma membrane–targeting TRPML1 mutant (TRPML1-4A) was overexpressed in HEK293 cells and the channel activities were directly measured by patching the plasma membrane. The sample traces for the current–voltage (I–V) curves of macroscopic currents shown in each figure were obtained from recordings on the same patch. All data points are mean ± SEM (*n* ≥ 5).

Detailed methods of protein purification, structure determination, and electrophysiology are provided in *SI Appendix*, *Materials and Methods*.

## Supplementary Material

Supplementary File

Supplementary File

Supplementary File

Supplementary File

Supplementary File

Supplementary File

Supplementary File

Supplementary File

## Data Availability

The cryo-EM density maps reported in this article of mouse TRPML1 have been deposited in the Electron Microscopy Data Bank under accession nos. 25379 (apo), 25378 [PI(3,5)P_2_-bound], 25380 [PI(3,5)P_2_/Tem-bound], and 25377 (ML-SA1–bound). Atomic coordinates have been deposited in the PDB under ID codes 7SQ8 (apo), 7SQ7 [PI(3,5)P_2_-bound], 7SQ9 [PI(3,5)P_2_/Tem-bound], and 7SQ6 (ML-SA1–bound).
