# Supplementary material for: Structural mechanism of allosteric activation of TRPML1 by PI(3,5)P2 and rapamycin
Source: Proc Natl Acad Sci U S A. 2022 Feb 7;119(7):e2120404119. doi: 10.1073/pnas.2120404119 (PMC8851561; doi:10.1073/pnas.2120404119)
Supplement: Supplementary File [file pnas.2120404119.sapp.pdf]

Supplementary Information for:

Structural mechanism of allosteric activation of TRPML1 by PI(3,5)P_2 and rapamycin

Ninghai Gan[1,2], Yan Han[1,2], Weizhong Zeng[1,2], Yan Wang[1,2], Jing Xue[1,2] & Youxing Jiang[1,2]

Affiliations:

[1] Howard Hughes Medical Institute and Department of Physiology, University of Texas Southwestern Medical Center, Dallas, Texas, USA

[2] Department of Biophysics, University of Texas Southwestern Medical Center, Dallas, Texas, USA

[*]Correspondence to:

Youxing Jiang, Ph.D., Department of Physiology, UT Southwestern Medical Center, 5323 Harry Hines Blvd., Dallas, Texas 75390-9040, Tel. 214 645-6027; Fax. 214 645-6042; E-Mail: youxing.jiang@utsouthwestern.edu

This PDF file includes:

Supplementary Materials and Methods

Supplementary Fig. S1-S7

Supplementary Table S1

Supplementary Movie Legends

Supplementary References

Protein expression and purification

The *Mus musculus* TRPML1 gene with a C-terminal thrombin cleavage site and a 10X His tag was cloned into a pEZTBM vector[1] and heterologously expressed in HEK293F cells using the BacMam system. The baculovirus was produced in Sf9 cells and used to transduce the HEK293F cells at a ratio of 1:40 (virus:HEK293F, v/v) and supplemented with 1 mM sodium butyrate to boost the protein expression. Cells were cultured in suspension at 37 °C for 48 h and harvested by centrifugation at 3,000g. All purification procedures were carried out at 4 °C unless specified otherwise. The cell pellet was re-suspended in buffer A (20 mM Tris pH 8.0, 150mM NaCl) supplemented with a protease inhibitor cocktail (containing 1 mg/ml each of DNase, pepstatin, leupeptin, and aprotinin and 1 mM PMSF) and homogenized by sonication on ice. Protein was extracted with 1% (w/v) n-dodecyl-β-D-maltopyranoside (DDM; Anatrace) supplemented with 0.2% (w/v) cholesteryl hemisuccinate (CHS; Sigma-Aldrich) or 1% (w/v) Lauryl Maltose Neopentyl Glycol (LMNG; Anatrace) by gentle agitation for 2 h. After extraction, the supernatant was collected after a 1 h centrifugation at 48,000g and incubated with Ni-NTA resin and 20 mM imidazole with gentle agitation. After 1 h, the resin was collected on a disposable gravity column (Bio-Rad), washed with buffer B (buffer A + 0.04%glyco-diosgenin (GDN; Anatrace)) with 20 mM imidazole. The washed resin was left on-column in buffer B and digested with thrombin overnight. After digestion, the flow-through was concentrated and purified by size-exclusion chromatography on a Superose 6 10/300 GL column (GE Healthcare) pre-equilibrated with buffer B. The protein peak was collected and concentrated. For PI(3,5)P_2 -bound structure, the purified protein was incubated with 0.3mM PI(3,5)P_2 or 0.3mM PI(3,5)P_2/0.5mM Tem, for PI(3,5)P_2/Tem-bound structure, the protein was incubated with 0.3mM PI(3,5)P_2 and 0.5mM Tem, for ML-SA1-bound structure, the protein was incubated with 0.3mM ML-SA1. The Apo closed state is derived from the sample treated with 0.5mM Tem (Table S1). All samples were diluted to a 3.5mg/mL final concentration and incubated on ice for 4 h. PI(3,5)P_2 diC8 (Echelon) was used as the lipid ligand for both structural and functional analyses in this study.

Electron microscopy data acquisition

The cryo-EM grids were prepared by applying 3.5 µl protein (3.5 mg/mL) to a glow-discharged Quantifoil R1.2/1.3 200-mesh copper holey carbon grid (Quantifoil, Micro Tools GmbH) and blotted for 3.0 s under 100% humidity at 4 °C before being plunged into liquid ethane using a Mark IV Vitrobot (FEI). Micrographs were acquired on a Titan Krios microscope (FEI) operated at 300 kV with a K3 Summit direct electron detector (Gatan), using a slit width of 20 eV on a GIF-Quantum energy filter. Data were collected using the CDS (Correlated Double Sampling) mode of the K3 camera with a super-resolution pixel size of 0.415 Å (for the TRPML1 sample prepared in the presence of PI(3,5)P_2 dataset, the pixel size is 0.4235 Å). The defocus range was set from −0.9 to −2.2 µm. Each movie was dose-fractionated to 60 frames with a dose rate of 1e^-/Å^2/frame for a total dose of 60e^-/Å^2. The total exposure time was between 5 to 6 s.

Image processing

Movie frames were motion-corrected and binned two times and dose-weighted using MotionCor2[2]. The CTF parameters of the micrographs were estimated using the GCTF program[3]. The rest of the image processing steps were carried out using RELION 3.1[4-6]. All resolution was reported according to the gold-standard Fourier shell correlation (FSC) using the 0.143 criterion[7]. Local resolution was estimated using Relion. Aligned micrographs were manually inspected to remove those with ice contamination and bad defocus. Particles were selected using Gautomatch (K. Zhang, MRC LMB, https://www2.mrc-lmb.cam.ac.uk/research/locally-developed-software/zhang-software/) and extracted using a binning factor of 3. 2D classification was performed in Relion 3.1. Selected particles after 2D classification were subjected to one round of 3D classification. The mouse TRPML1 map (EMD-8883[8]) low-pass filtered to 30 Å was

used as the initial reference. Classes that showed clear features of the TRPML1 channel were combined and subjected to 3D auto-refinement and another round of 3D classification without performing particle alignment using a soft mask around the protein portion of the density. C4 symmetry was imposed in these steps. The best resolving classes were then re-extracted with the original pixel size and further refined. Beam tilt, anisotropic magnification, and per-particle CTF estimations, and Bayesian polishing were performed in Relion 3.1 to improve the resolution of the final reconstruction.

For the dataset of apo structure using the protein sample prepared in the presence of Tem alone, a total of 4,929 movies were collected and 4,745 were selected after motion correction and CTF estimation. A total number of 753,663 particles were extracted from the selected micrographs and were subjected to one round of 2D classification, from which 147,094 particles were selected. After the initial 3D classification, 47,179 particles were selected and subjected to a 3D auto-refinement job. Next, a soft mask excluding the micelle density was applied and particles were sorted into 4 classes without performing the alignment. From this, one class with 33,553 particles was selected and further refined, yielding a map at 2.6Å overall resolution (Figure S2).

For the dataset of PI(3,5)P_2-bound structure obtained using the protein sample prepared in the presence of PI(3,5)P_2 alone, a total of 6,498 movies were collected and 6,474 were selected after motion correction and CTF estimation. A total number of 1,150,065 particles were extracted from the selected micrographs and were subjected to one round of 2D classification, from which 469,210 particles were selected. After the initial 3D classification, 139,005 particles were selected and subjected to a 3D auto-refinement job, yielding a map at 2.6Å overall resolution (Figure S3).

For the dataset of protein sample prepared in the presence of PI(3,5)P_2 and Tem, a total of 5,071 movies were collected and 4,791 were selected after motion correction and CTF estimation. A total number of 1,262,471 particles were extracted from the selected micrographs and were subjected to one round of 2D classification, from which 638,713 particles were selected. After the initial 3D classification, 266,346 particles were selected and subjected to a 3D auto-refinement job. Next, a soft mask excluding the micelle density was applied and particles were sorted into 5 classes without performing the alignment. From this, two conformations of the channel with 67,182 and 142,902 particles for PI(3,5)P_2-bound and PI(3,5)P_2/Tem-bound states, respectively, were selected for further refinement. The particles of PI(3,5)P_2-bound class were then classified with a soft mask around the PI(3,5)P_2 ligand density with regularisation parameter T=8, and 53,514 particles were selected from this classification. These particles were then refined and yielded a density map at an overall resolution of 2.4 Å. The particles of PI(3,5)P_2/Tem-bound class were further refined in Relion and yielded a map with an overall resolution of 2.1 Å (Figure S4).

For the dataset of ML-SA1-bound structure, a total of 5,163 movies were collected and 5,061 were selected after motion correction and CTF estimation. A total number of 831,409 particles were extracted from the selected micrographs and were subjected to one round of 2D classification, from which 309,621 particles were selected. After the initial 3D classification, 164,721 particles were selected and subjected to a 3D auto-refinement job. Next, a soft mask excluding the micelle density was applied and particles were sorted into 5 classes without performing the alignment. From this, two classes (with a total number of 41,980 particles) of the channel were selected and further refined, yielding a map at 2.3Å overall resolution (Figure S5).

Model building, refinement, and validation

The structure of mouse TRPML1 (PDB code: 5WPV) was used as the initial model and was manually adjusted in Coot[9] and refined against the map by using the real-space refinement module with secondary structure and non-crystallographic symmetry restraints in the Phenix package[10]. The final structure models include residues 40-198, 215-285 (or 215-291), and 296-527. About 40 residues at the amino terminus and 50 residues at the carboxy terminus are disordered and not modeled. The statistics of the geometries of the models were generated using MolProbity[11]. All the figures were prepared in PyMol (Schrödinger, LLC.), UCSF Chimera[12]. Pore radii were calculated using the HOLE program[13].

Electrophysiology

The N-terminal GFP tagged, plasma membrane-targeting TRPML1 mutant (TRPML1-4A)[14,15] was overexpressed in HEK293 cells and the channel activities were directly measured by patching the plasma membrane. In this setting, the extracellular side is equivalent to the luminal side of TRPML1 in endosomes or lysosomes. 48 h after transfection, cells were dissociated by trypsin treatment and kept in serum-containing complete medium; the cells were re-plated onto 35 mm tissue culture dishes and kept in a tissue culture incubator until recording. Patch clamp in the whole-cell or inside-out configuration was used to measure TRPML1 activity on the HEK plasma membrane. The standard bath solution for whole-cell current recording contained (in mM): 145 sodium methanesulfonate, 5 NaCl, 1 $MgCl_2$, 10 HEPES buffered with Tris, pH 7.4; and the pipette solution contained (in mM): 140 cesium methanesulfonate, 5 NaCl, 5 $MgCl_2$, 10 EGTA, 10 HEPES buffered with Tris, pH 7.4. The bath solution for inside-out configuration contained (in mM): 140 potassium methanesulfonate, 5 NaCl, 2 $MgCl_2$, 0.4 $CaCl_2$, 1 EGTA, 10 HEPES buffered with Tris, pH 7.4; and the pipette solution contained (in mM): 145 sodium methanesulfonate, 5 NaCl, 1 $MgCl_2$, 0.5 EGTA, 10 HEPES buffered with Tris, pH 7.4. For the whole-cell recording of PI(3,5)P_2-activated channel, we had to include a high concentration of PI(3,5)P_2 (100 μM) in the pipette solution (cytosolic side) to quickly obtain a stable PI(3,5)P_2-evoked current, likely because of the slow diffusion of this lipid ligand. The patch pipettes were pulled from Borosilicate glass and heat polished to a resistance of 2–5 MΩ (2–3 MΩ for inside-out patch, and 3–5 MΩ for whole-cell current recording). Data were acquired using an AxoPatch 200B amplifier (Molecular Devices) and a low-pass analog filter set to 1 kHz. The current signal was sampled at a rate of 20 kHz using a Digidata 1550B digitizer (Molecular Devices) and further analyzed with pClamp 11 software (Molecular Devices). After the patch pipette was attached to the cell membrane, the giga seal (>10 GΩ) was formed by gentle suction. The inside-out configuration was formed by pulling the pipette away from the cell, and the pipette tip was exposed to the air for 2 seconds. The whole-cell configuration was formed by a short zap or suction to rupture the patch. The holding potential was set to 0 mV. The whole-cell and inside-out macroscopic current recordings were obtained using voltage pulses ramped from −140 mV to +50 mV over 800 ms. The sample traces for the I–V curves of macroscopic currents shown in each figure were obtained from recordings on the same patch. All data points are mean ± s.e.m. ($n \geq 5$).

Data availability

The cryo-EM density maps of the mouse TRPML1 have been deposited in the Electron Microscopy Data Bank (EMDB) under accession numbers 25379 (Apo), 25378 (PI(3,5)P_2-bound), 25380 (PI(3,5)P_2/Tem-bound), 25377 (ML-SA1-bound). Atomic coordinates have been deposited in the Protein Data Bank (PDB) under accession numbers 7SQ8 (Apo), 7SQ7 (PI(3,5)P_2-bound), 7SQ9 (PI(3,5)P_2/Tem-bound), 7SQ6 (ML-SA1-bound).

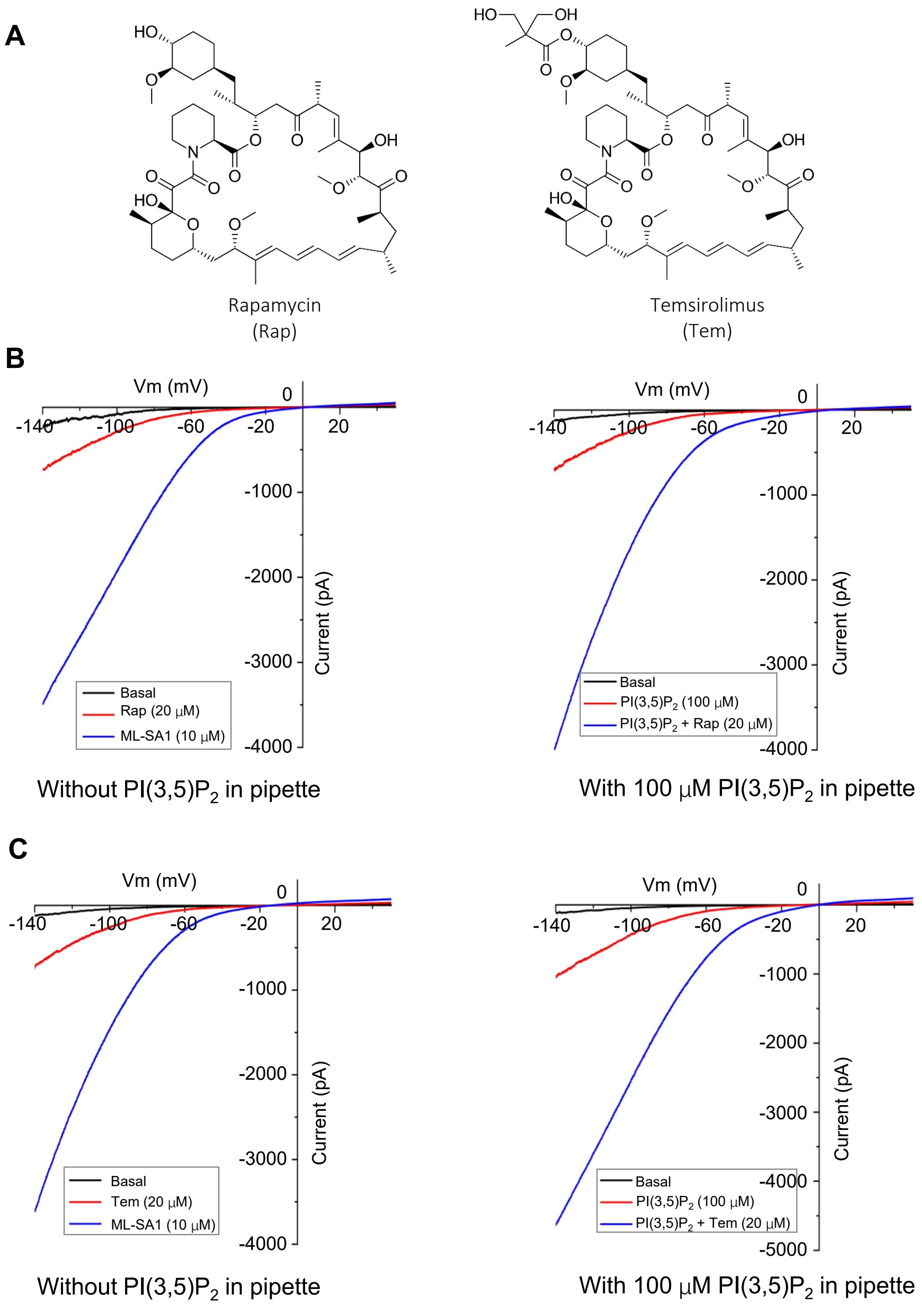

Figure S1: Structural and functional comparison between rapamycin and temsirolimus.

A. Chemical structures of rapamycin and temsirolimus.
B. Sample traces of TRPML1 activation recorded using patch clamp in whole-cell configuration with or without $PI(3,5)P_2$ (100 μM) in the pipette (cytosolic). Rapamycin and ML-SA1 (for comparison) were introduced in the bath solution (extracellular/luminal). Rapamycin elicit small inward current in the absence of $PI(3,5)P_2$ but a large current with the presence of $PI(3,5)P_2$ in pipette.
C. Sample traces of TRPML1 activation by temsirolimus recorded using patch clamp in whole-cell configuration with or without $PI(3,5)P_2$ (100 μM) in the pipette (cytosolic). Temsirolimus exhibits similar channel activation property as rapamycin.

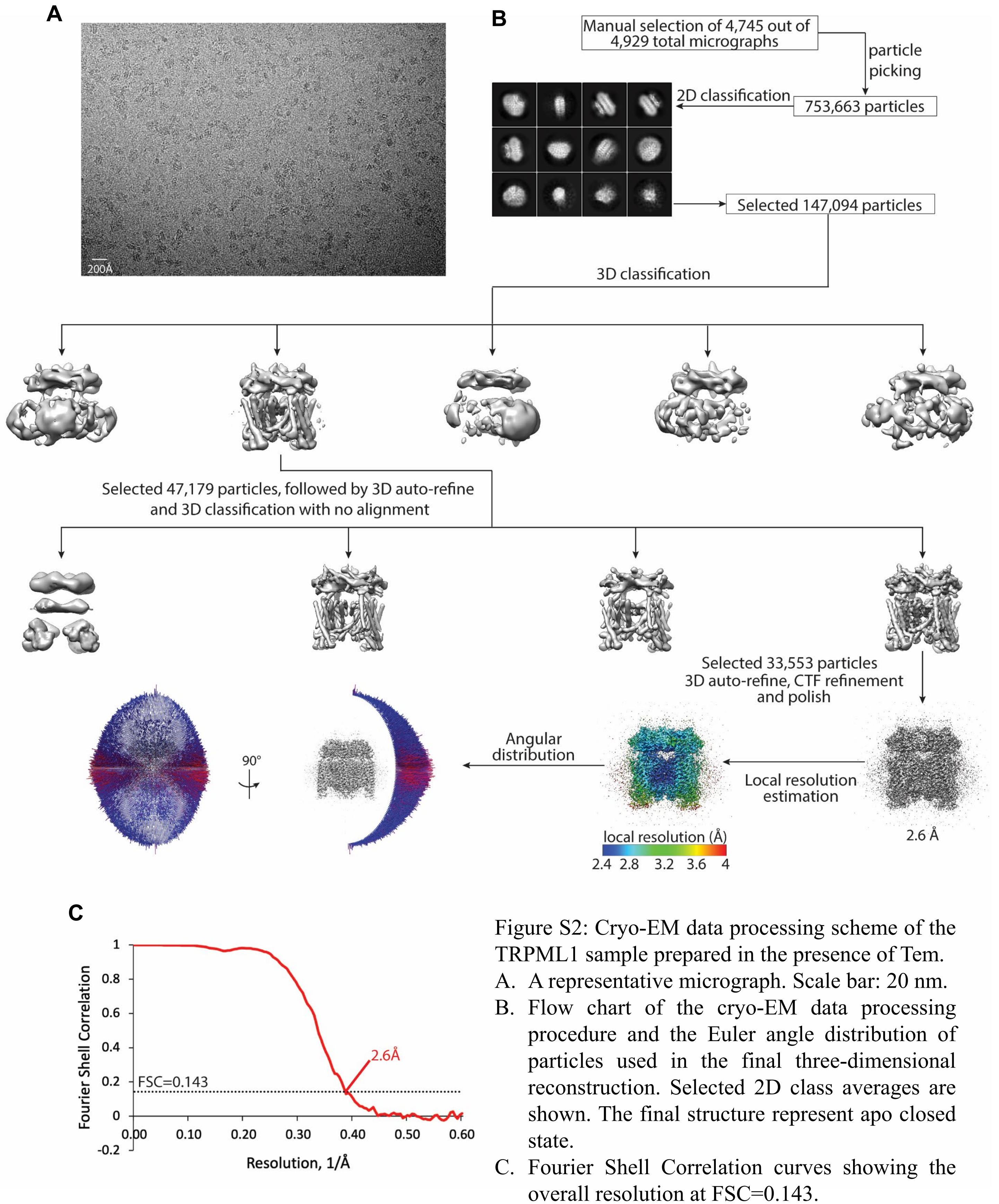

Figure S2: Cryo-EM data processing scheme of the TRPML1 sample prepared in the presence of Tem.

A. A representative micrograph. Scale bar: 20 nm.
B. Flow chart of the cryo-EM data processing procedure and the Euler angle distribution of particles used in the final three-dimensional reconstruction. Selected 2D class averages are shown. The final structure represent apo closed state.
C. Fourier Shell Correlation curves showing the overall resolution at FSC=0.143.

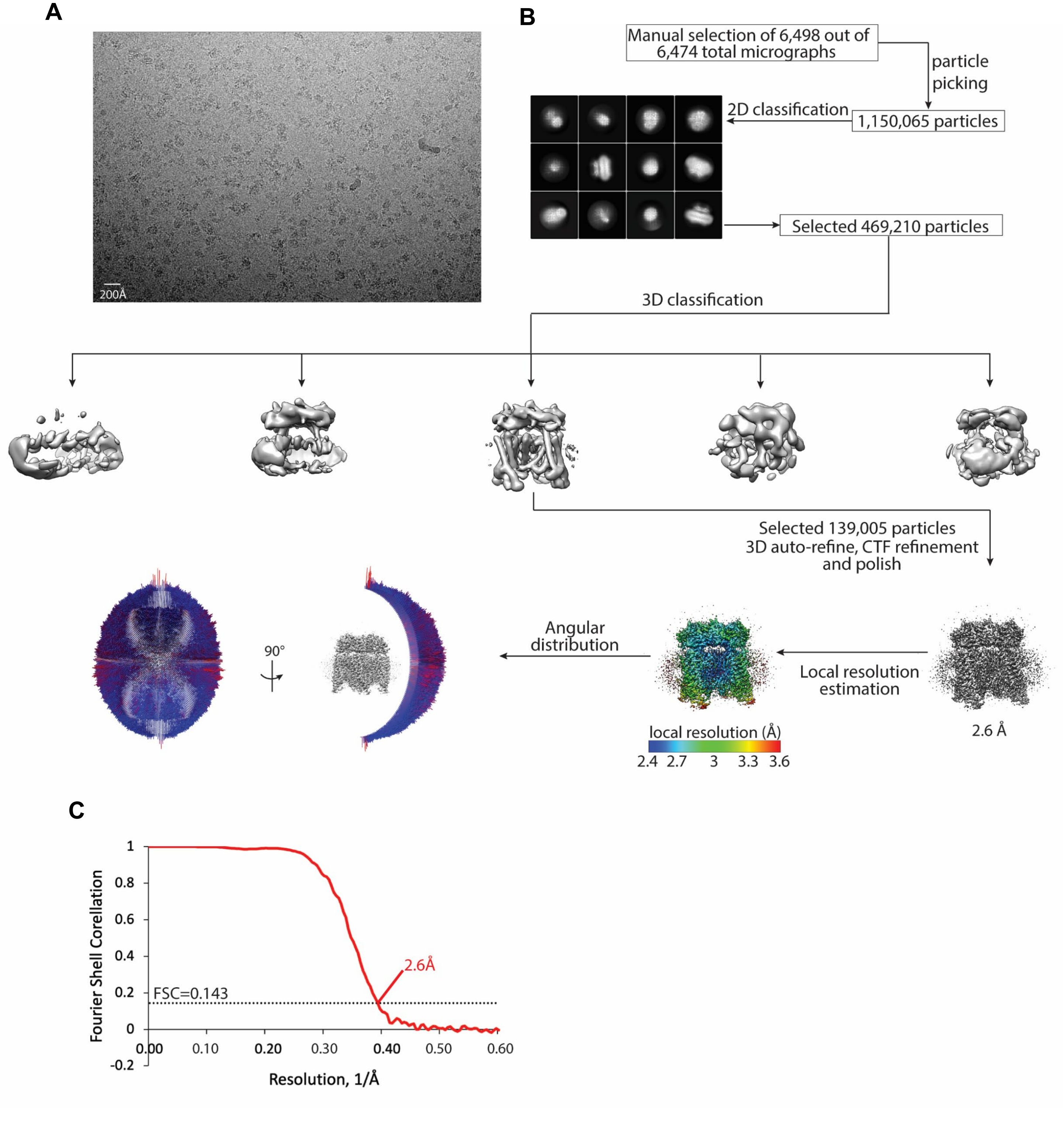

Figure S3: Cryo-EM data processing scheme of the TRPML1 sample prepared in the presence of $PI(3,5)P_2$.

A. A representative micrograph. Scale bar: 20 nm.
B. Flow chart of the cryo-EM data processing procedure and the Euler angle distribution of particles used in the final three-dimensional reconstruction. Selected 2D class averages are shown. The final structure represent $PI(3,5)P_2$-bound closed state.
C. Fourier Shell Correlation curves showing the overall resolution at FSC=0.143.

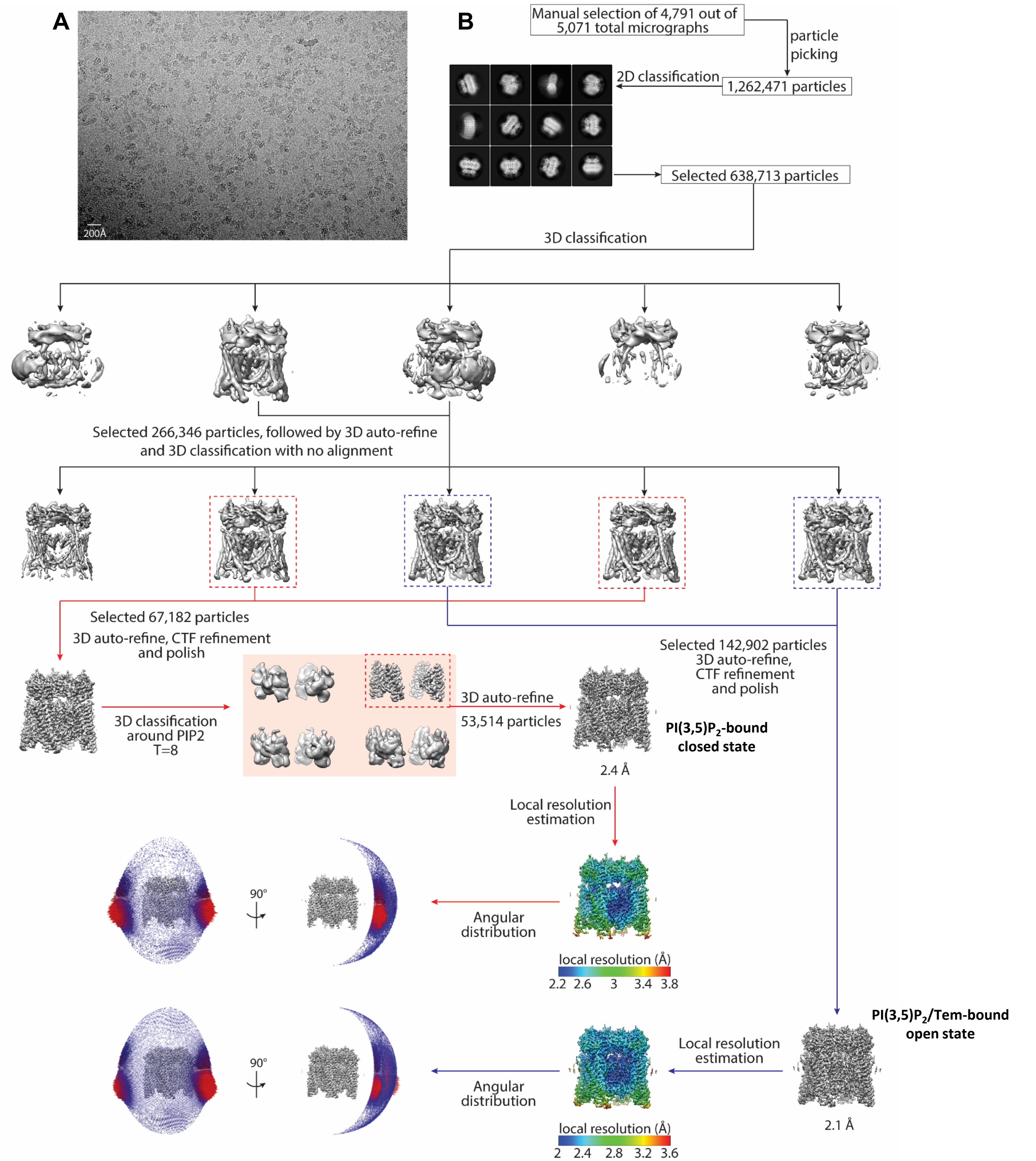

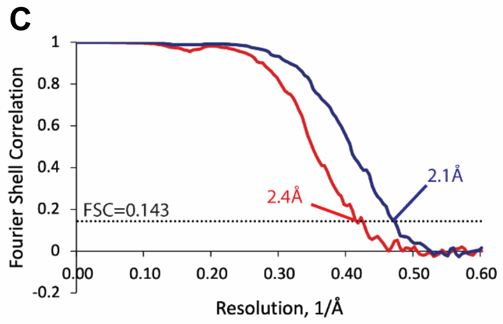

Figure S4: Cryo-EM data processing scheme of the TRPML1 sample prepared in the presence of PI(3,5)P_2 and Tem.

A. A representative micrograph. Scale bar: 20 nm.

B. Flow chart of the cryo-EM data processing procedure and the Euler angle distribution of particles used in the final three-dimensional reconstruction. Selected 2D class averages are shown. Two final structures represent PI(3,5)P_2-bound closed state and PI(3,5)P_2/Tem-bound open state, respectively.

C. Fourier Shell Correlation curves showing the overall resolution at FSC=0.143.

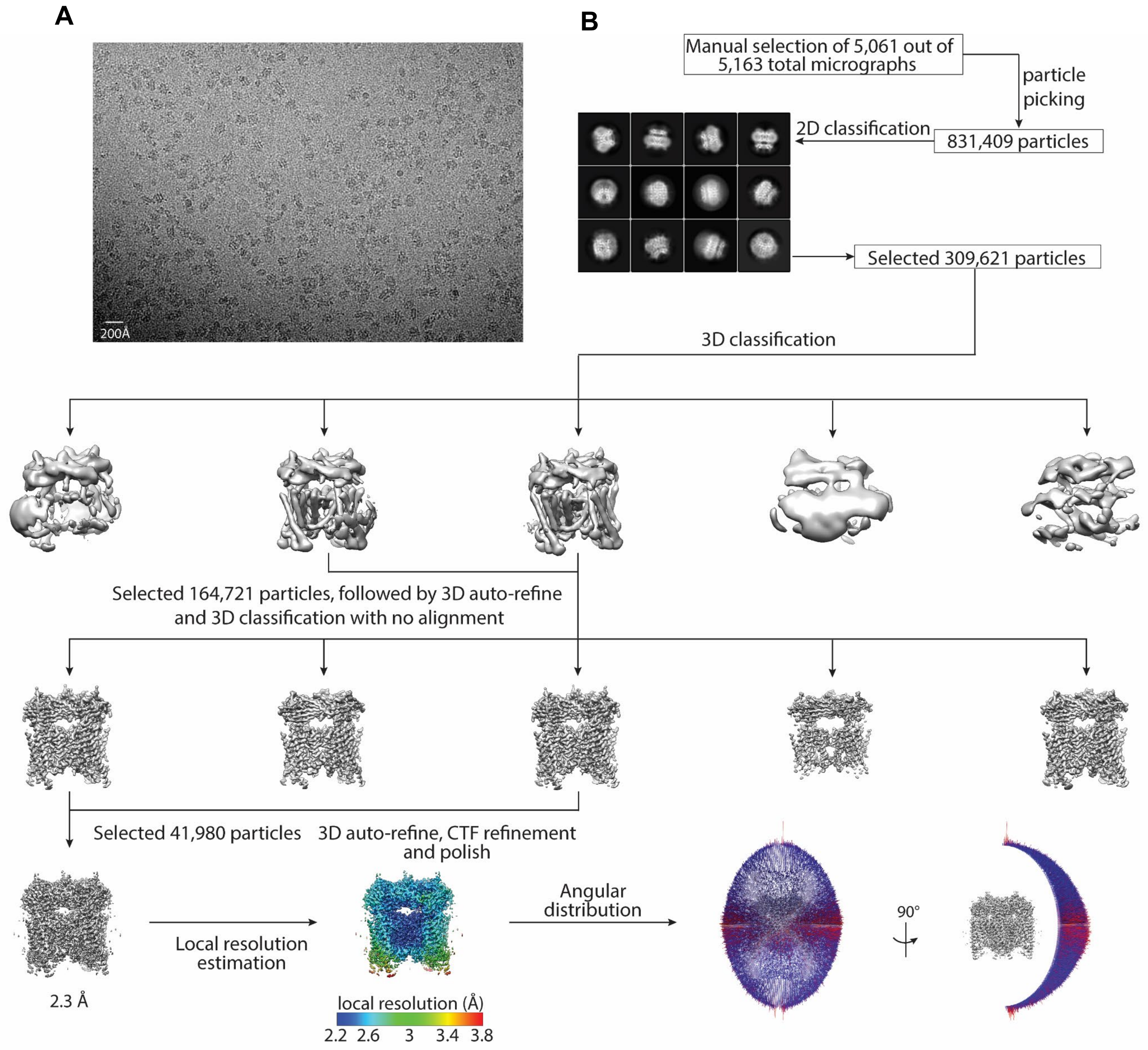

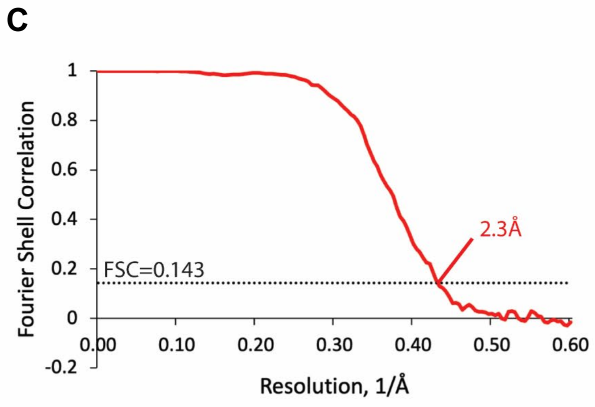

Figure S5: Cryo-EM data processing scheme of the TRPML1 sample prepared in the presence of ML-SA1.

A. A representative micrograph. Scale bar: 20 nm.
B. Flow chart of the cryo-EM data processing procedure and the Euler angle distribution of particles used in the final three-dimensional reconstruction. Selected 2D class averages are shown. The final structure represent ML-SA1-bound open state.
C. Fourier Shell Correlation curves showing the overall resolution at FSC=0.143.

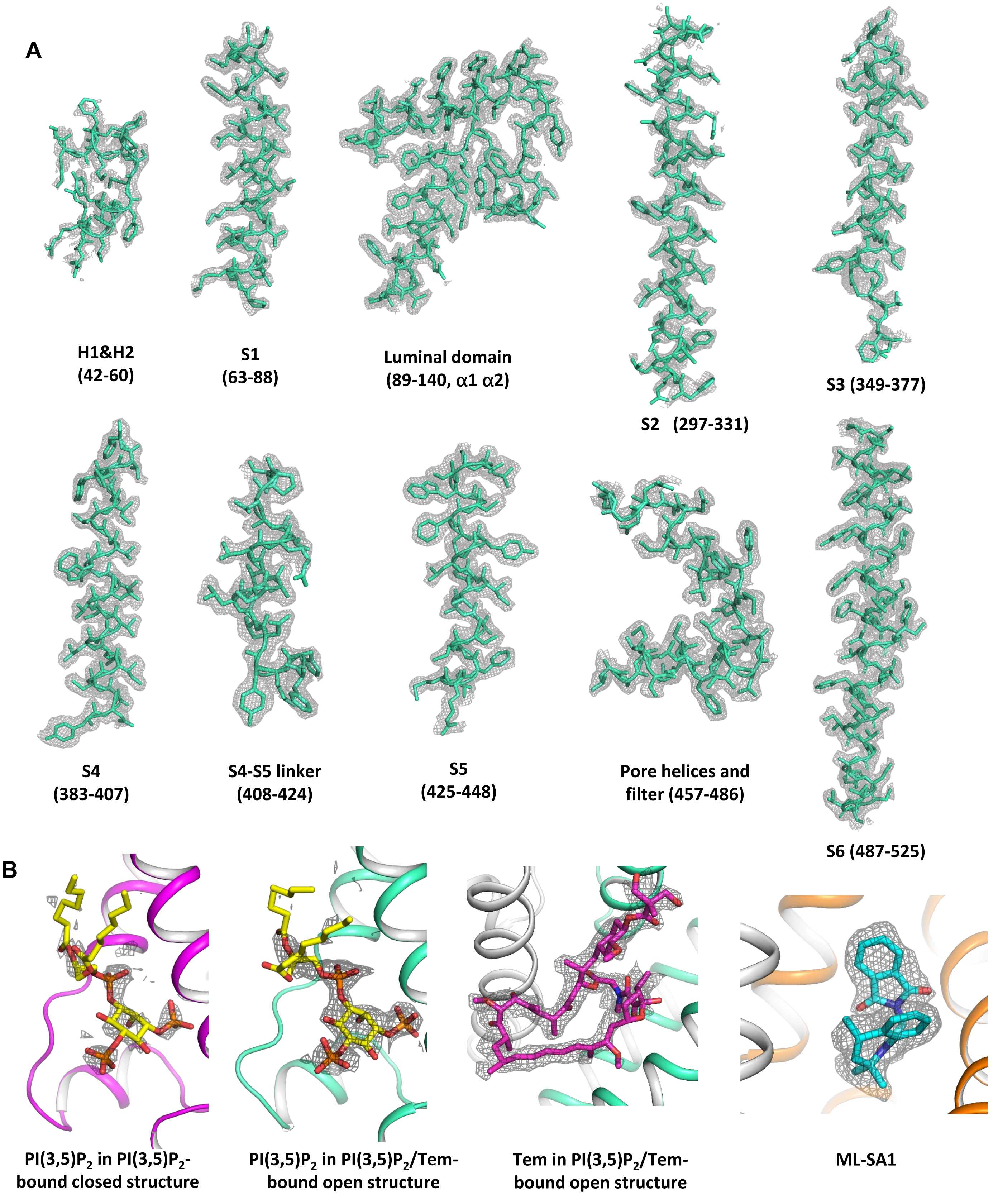

Figure S6: Sample density maps of TRPML1.
A. Maps of the PI(3,5)P_2/Tem-bound open structure contoured at 4 σ.
B. Maps of the bound ligands contoured at 4 σ.

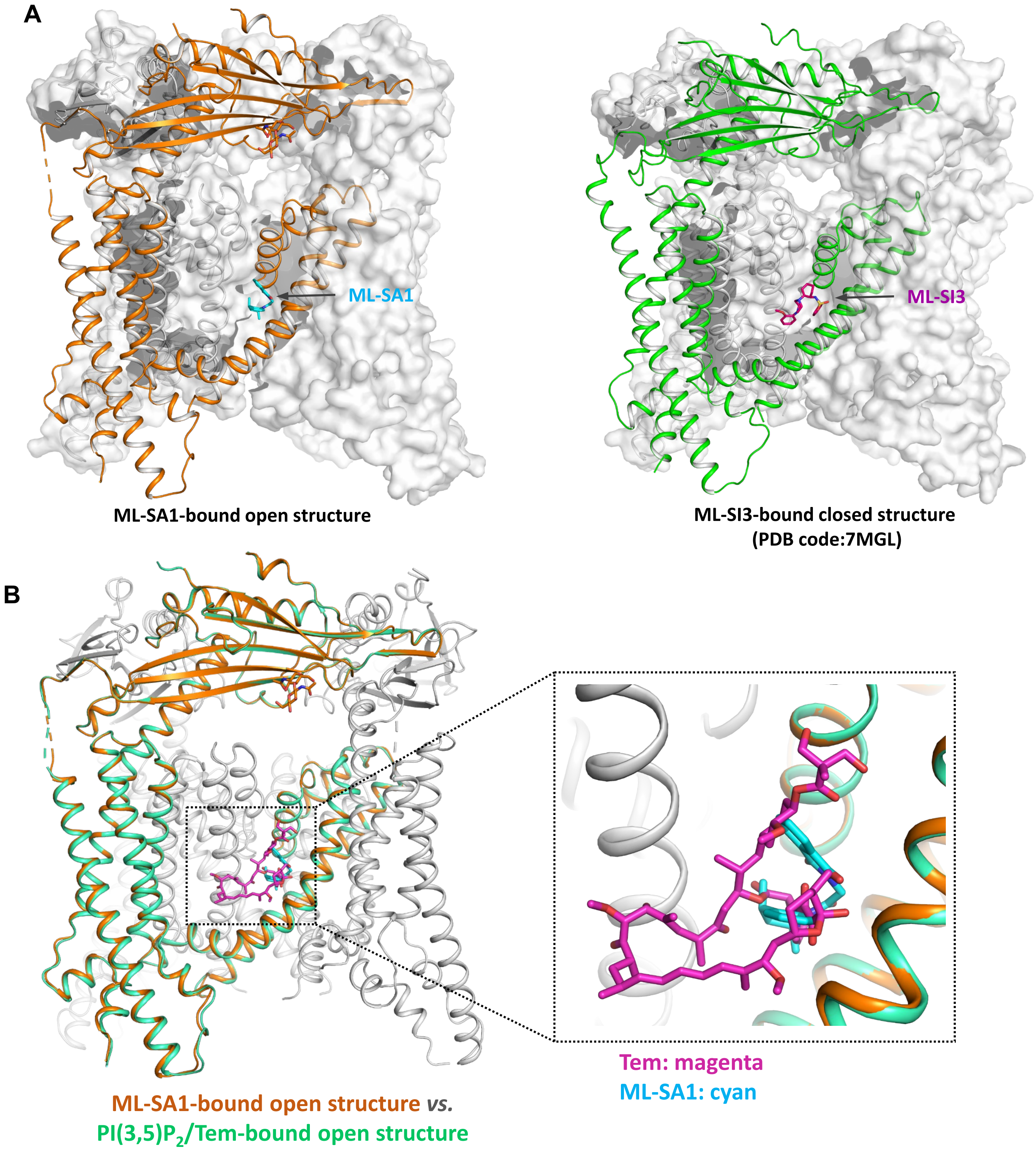

Figure S7: Comparison of Tem binding with synthetic agonist ML-SA1 and antagonist ML-SI3.

A. Structures of ML-SA1-bound TRPML1 (left, from this study) and ML-SI3-bound TRPML1 (right, PDB code: 7MGL).
B. Structural comparison between the ML-SA1-bound open structure (orange) and the PI(3,5)P_2/Tem-bound open structure (green). Inset: zoomed-in view of the overlapped binding site between Tem and ML-SA1.

Supplementary Table 1. Data collection and refinement statistics.

	**Apo TRPML1**<br>EMDB-25379<br>PDB-7SQ8	**$PI(3,5)P_2$-bound TRPML1**<br>EMDB-25378<br>PDB-7SQ7	**$PI(3,5)P_2$/Tem-bound TRPML1**<br>EMDB-25380<br>PDB-7SQ9	**ML-SA1-bound TRPML1**<br>EMDB-25377<br>PDB-7SQ6
Ligands in sample preparation	0.5mM Tem	0.3mM $PI(3,5)P_2$ /0.5mM Tem	0.3mM $PI(3,5)P_2$ /0.5mM Tem	0.3mM ML-SA1
**Data collection and processing**				
Magnification	105,000	105,000	105,000	105,000
Voltage (kV)	300	300	300	300
Electron exposure (e–/Å2)	60	60	60	60
Defocus range (μm)	-0.9 - -2.2	-0.9 - -2.2	-0.9 - -2.2	-0.9 - -2.2
Pixel size (Å)	0.83	0.83	0.83	0.83
Symmetry imposed	C4	C4	C4	C4
Initial particle images (no.)	753,663	1,262,471	1,262,471	831,409
Final particle images (no.)	33,553	67,182	142,902	41,980
Map resolution (Å)	2.59	2.41	2.11	2.32
FSC threshold	0.143	0.143	0.143	0.143
**Refinement**				
Initial model used (PDB code)	5WPV	5WPV	5WPV	5WPV
Model resolution (Å) FSC threshold	2.59	2.41	2.11	2.32
Map sharpening B factor (Å2)	-54.70	-46.87	-39.23	-42.84
Model composition				
Non-hydrogen atoms	15109	15473	15,970	15418
Protein residues	1852	1852	1,876	1868
Ligands	9	13	18	14
B factors (Å2)				
Protein	37.23	46.01	32.02	40.94
Ligand	43.38	59.34	38.97	31.26
R.m.s. deviations				
Bond lengths (Å)	0.003	0.007	0.005	0.006
Bond angles (°)	0.534	0.854	0.999	0.764
Validation				
MolProbity score	1.36	1.53	1.31	1.39
Clashscore	3.68	5.00	5.11	4.73
Poor rotamers (%)	0	0	0	0
Ramachandran plot				
Favored (%)	96.72	96.06	97.84	97.18
Allowed (%)	3.28	3.94	2.16	2.82
Disallowed (%)	0	0	0	0

Supplementary Movies:

Movie S1: Top view of pore opening.

Movie S2: Side view of pore opening. Only two diagonal subunits are shown for clarity.

Movie S3: Conformational changes at Tem-binding site.

Movie S4: Movement 1 at PI(3,5)P_2-binding site.

Movie S5: Movement 2 (S4 bending) at PI(3,5)P_2-binding site.

Movie S6: Overall view of TRPML1 global conformational changes from closed to open state.

Movie S7: Top view of TRPML1 showing the rotation of luminal linker domain from closed to open state.

Supplementary References:

1 Morales-Perez, C. L., Noviello, C. M. & Hibbs, R. E. Manipulation of Subunit Stoichiometry in Heteromeric Membrane Proteins. *Structure* **24**, 797-805, doi:10.1016/j.str.2016.03.004 (2016).

2 Zheng, S. Q. *et al.* MotionCor2: anisotropic correction of beam-induced motion for improved cryo-electron microscopy. *Nat Methods* **14**, 331-332, doi:10.1038/nmeth.4193 (2017).

3 Zhang, K. Gctf: Real-time CTF determination and correction. *J Struct Biol* **193**, 1-12, doi:10.1016/j.jsb.2015.11.003 (2016).

4 Nakane, T. *et al.* Single-particle cryo-EM at atomic resolution. *Nature* **587**, 152-156, doi:10.1038/s41586-020-2829-0 (2020).

5 Scheres, S. H. RELION: implementation of a Bayesian approach to cryo-EM structure determination. *J Struct Biol* **180**, 519-530, doi:10.1016/j.jsb.2012.09.006 (2012).

6 Zivanov, J. *et al.* New tools for automated high-resolution cryo-EM structure determination in RELION-3. *Elife* **7**, doi:10.7554/eLife.42166 (2018).

7 Henderson, R. *et al.* Outcome of the first electron microscopy validation task force meeting. *Structure* **20**, 205-214, doi:10.1016/j.str.2011.12.014 (2012).

8 Chen, Q. *et al.* Structure of mammalian endolysosomal TRPML1 channel in nanodiscs. *Nature* **550**, 415-418, doi:10.1038/nature24035 (2017).

9 Emsley, P., Lohkamp, B., Scott, W. G. & Cowtan, K. Features and development of Coot. *Acta Crystallogr D Biol Crystallogr* **66**, 486-501, doi:10.1107/s0907444910007493 (2010).

10 Adams, P. D. *et al.* PHENIX: a comprehensive Python-based system for macromolecular structure solution. *Acta Crystallogr D Biol Crystallogr* **66**, 213-221, doi:10.1107/s0907444909052925 (2010).

11 Chen, V. B. *et al.* MolProbity: all-atom structure validation for macromolecular crystallography. *Acta Crystallogr D Biol Crystallogr* **66**, 12-21, doi:10.1107/s0907444909042073 (2010).

12 Pettersen, E. F. *et al.* UCSF Chimera--a visualization system for exploratory research and analysis. *J Comput Chem* **25**, 1605-1612, doi:10.1002/jcc.20084 (2004).

13 Smart, O. S., Neduvelil, J. G., Wang, X., Wallace, B. A. & Sansom, M. S. HOLE: a program for the analysis of the pore dimensions of ion channel structural models. *J Mol Graph* **14**, 354-360, 376, doi:10.1016/s0263-7855(97)00009-x (1996).

14 Vergarajauregui, S. & Puertollano, R. Two di-leucine motifs regulate trafficking of mucolipin-1 to lysosomes. *Traffic* **7**, 337-353, doi:10.1111/j.1600-0854.2006.00387.x (2006).

15 Grimm, C. *et al.* Small molecule activators of TRPML3. *Chem Biol* **17**, 135-148, doi:10.1016/j.chembiol.2009.12.016 (2010).
